# Bleomycin Sensitivity in *Escherichia coli* is Medium-Dependent

**DOI:** 10.1371/journal.pone.0033256

**Published:** 2012-03-15

**Authors:** Tao Xu, William Brown, Martin G. Marinus

**Affiliations:** Department of Biochemistry and Molecular Pharmacology, University of Massachusetts Medical School, Worcester, Massachusetts, United States of America; University of Massachusetts Medical School, United States of America

## Abstract

Bleomycin (BLM) is a glycopeptide antibiotic and anti-tumor agent that targets primarily the furanose rings of DNA and in the presence of ferrous ions produces oxidative damage and DNA strand breaks. *Escherichia coli* cells growing in broth medium and exposed to low concentrations of BLM contain double-strand breaks and require homologous recombination to survive. To a lesser extent, the cells also require the abasic (AP) endonucleases associated with base excision repair, presumably to repair oxidative damage. As expected, there is strong induction of the SOS system in treated cells. In contrast, *E. coli* cells growing in glucose or glycerol minimal medium are resistant to the lethal action of BLM and do not require either homologous recombination functions or AP-endonucleases for survival. DNA ligase activity, however, is needed for cells growing in minimal medium to resist the lethal effects of BLM. There is weak SOS induction in such treated cells.

## Introduction

Bleomycin (BLM) is a glycopeptide antibiotic and anti-tumor agent isolated from *Streptomyces verticillis* [1], [2] that targets primarily the furanose rings of DNA. Degradation by BLM is initiated by generating a free radical, in the presence of ferrous ion, in the deoxyribose resulting in two different types of DNA damage [3], [4]. At low oxygen tension, oxidized abasic (AP) sites are favored while at high oxygen tension single-and double-strand breaks (DSBs) predominate. These alternative pathways lead to a mix of abasic sites and strand breaks which occur at a 1∶1 ratio [5]. The DSBs are suspected to be the major cause of cell death. Up to one-third of BLM-induced lesions are double-strand breaks which consist of either two identical breaks in opposite strands or arise from an abasic site with a closely opposed strand break [6].

In order to more fully understand the mechanism of BLM toxicity in *Escherichia coli*, previous studies [7], [8] have shown an increased BLM sensitivity of *lexA* and *recA* mutant strains indicating that the SOS response is an important mechanism cells use to resist the toxic effects of the drug. The increased sensitivity of *recA* and *recBC* mutant strains to BLM indicates a role for homologous recombination in cell survival [8], [9]. This result is consistent with a requirement for recombinational repair as a consequence of DSBs in DNA. Surprisingly, *recN* and *recG* mutant strains, in an otherwise wildtype background, were also found to be sensitive to BLM exposure, a feature not shared with other common DNA damaging agents [8]. The role of RecN in recombination/repair is unknown while RecG is a helicase that translocates Holliday junction intermediates in recombination and repair [10].

We have confirmed and extended the above observations to additional mutant strains and show that at the low concentrations of BLM used here, homologous recombination is the principal pathway of repair in cells growing in broth. We also show that there is differential sensitivity of *E. coli* in glucose minimal medium versus rich medium after exposure to BLM.

## Results

### Strain construction

A previous study [8] used mutations affecting repair/recombination in an AB1157 background. These mutations are either transposon insertions or point mutations, many of which have not been characterized at the DNA sequence level. We have constructed a panel of mutant strains in a different genetic background (GM7330) using deletion alleles wherever possible and have used them in this study. These strains have been used previously to study the toxic effects of cisplatin and MNNG [11], [12].

### DNA repair-deficient mutant strains

**Figure 1:**
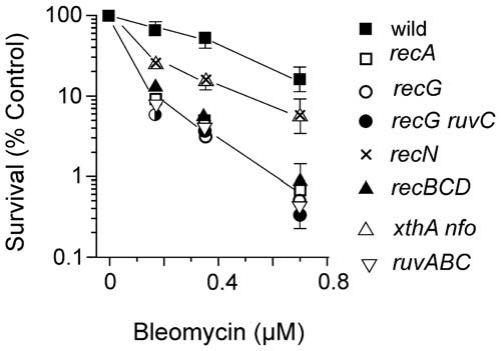
Survival of *E. coli* strains after exposure to BLM. Cells in the logarithmic phase of growth in L broth at 37°C were exposed to various concentrations of BLM for 30 min, and then diluted and plated on L medium to determine survival.

An *xthA nfo* strain, defective for AP endonucleases, was more sensitive than wildtype after BLM treatment (Fig. 1). This result is consistent with the known base damage inflicted by BLM requiring base excision repair enzymes. The Fpg and MutY glycosylases are active on oxidative base lesions but *fpg* (*mutM*) and *fpg mutY* mutant strains were not sensitive suggesting that some other base damage occurs that requires AP endonuclease action. Bacteria defective for nucleotide excision repair (*uvrA*) were as resistant as wildtype to the cytotoxic effects of BLM as were *dam* and *mutS* strains affected for mismatch repair (data not shown). Two temperature-sensitive mutant strains defective in DNA ligase (*lig-4* and *lig-7*) were more sensitive at the higher temperature when exposed to BLM (see below).

### Mutant strains affecting homologous recombination

We confirmed the previous observations [8] that *recA* and *recBCD* strains growing in L broth were more sensitive after BLM exposure than the wild type strain (Fig. 1). We also confirmed the increased sensitivity of *recN* and *recG* mutants relative to wildtype [8]. Bacteria deleted for the *ruvABC* genes (Holliday junction translocation and resolution) were among the most sensitive to BLM action (Fig. 1). However, *recF*, *recO*, *recR*, *sbcDC*, *recJ* and *recQ* mutant alleles did not affect survival to BLM relative to wildtype (data not shown). Double mutants bearing *recBCD* with *recF* or *recG* or *recQ* were no more sensitive to BLM than the *recBCD* strain (data not shown). Similarly, the *recG ruvC* mutant had the same survival to BLM as the *recG* mutant (Fig. 1).

### Survival in minimal versus rich medium

**Figure 2:**
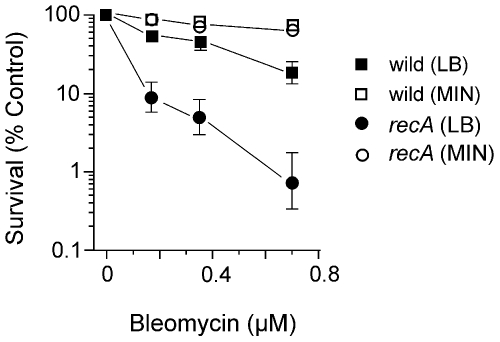
Survival of wildtype and *recA* cells to BLM in L broth and glucose minimal medium. Logarithmic phase cells growing at 37°C in L broth or glucose minimal medium were exposed to various concentrations of BLM for 30 min, and then diluted and plated on L medium to determine survival.

**Figure 3:**
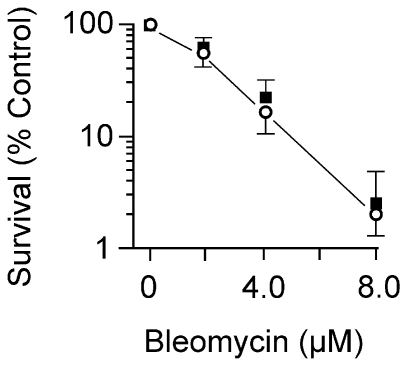
Survival of wildtype and *recA* cells to high concentrations of BLM. Logarithmic phase cells growing at 37°C in glucose minimal medium were exposed to various concentrations of BLM for 30 min, and then diluted and plated on L medium to determine survival. The BLM concentrations used in the Figure are ten-times greater than those used in Figures 1 and 2.

The survival experiments described above utilized wildtype and mutant cells grown in L broth. However, when wildtype cells were grown and exposed to BLM in glucose or glycerol minimal medium there was greater survival compared to the same doses used with wildtype cells in L broth (Fig. 2). The increased survival in glucose minimal versus L medium, was seen with all the mutant strains involved in recombination that were tested above (see Fig. 2 for the *recA* strain survival) and the *xthA nfo* AP-endonuclease-deficient strain (data not shown). At high BLM concentrations, there was increased killing of the wildtype cells (Fig. 3); however, even at these high concentrations the survival of the *recA* mutant strain paralleled that of the wildtype (Fig. 3). This result suggests that homologous recombination and SOS induction are not required to resist the toxic effects of BLM at these high concentrations.

**Figure 4:**
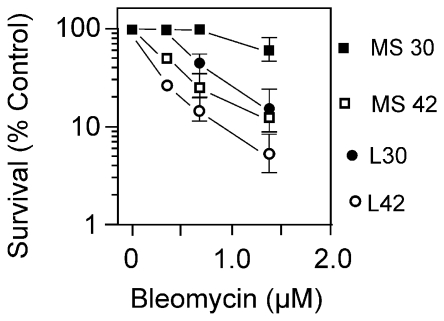
Survival of *lig-7* (Ts) cells to BLM in L broth and glucose minimal medium. Logarithmic phase *lig-7* (Ts) cells in L broth or glucose minimal medium at 30°C (permissive) or 42°C (non-permissive) were exposed to various concentrations of BLM for 30 min, and then diluted and plated on L medium to determine survival. The plates were incubated at 30°C.

Only the temperature sensitive DNA ligase-deficient strains showed sensitivity to BLM when cultivated in glucose minimal medium (Fig. 4). In L broth, reduced survival of the *lig-7* strain was apparent even at the permissive temperature and was decreased further at the non-permissive temperature. In glucose minimal medium, survival at both temperatures was higher compared to L broth. The survival at the permissive temperature was identical to a wildtype strain but there was a sharp decrease in survival at the non-permissive temperature (Fig. 4). The *lig-4* strain was also tested and gave qualitatively the same result as the *lig-7* strain (data not shown).

BLM requires ferrous ions to generate radicals but supplementing the glucose minimal medium with 1 µg/ml Fe_2_SO_4_ did not alter survival of wildtype or *recA* bacteria. L broth addition to glucose minimal medium in a 1∶1 ratio only partially reduced survival of wildtype cells (data not shown). Supplementing the glucose minimal medium with casamino acids (0.2%) did not change the survival compared to glucose minimal medium (data not shown). Supplementing L broth with individual components of glucose minimal medium did not produce an increase in survival (data not shown). Replacement of glucose with glycerol in the minimal medium did not alter survival (data not shown).

### Survival of cells growing in minimal medium to MNNG

**Figure 5:**
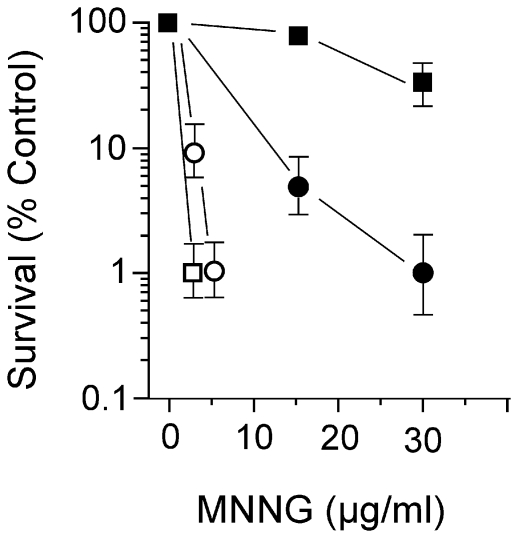
Survival of wildtype and *recA* cells to MNNG in L broth and glucose minimal medium. Logarithmic phase wildtype (filled symbols) or *recA* (open symbols) cells growing at 37°C in L broth (squares) or glucose minimal medium (circles) were exposed to various concentrations of MNNG for 30 min, and then diluted and plated on L medium to determine survival.

The surprising result that *E. coli* cells growing in glucose minimal medium were resistant to the lethal effects of BLM, prompted us to test another cytotoxic chemical – N-methyl-N′-nitro-N-nitrosoguanidine (MNNG). MNNG was chosen because its action results in the formation of methylated bases that are subject to base excision repair and that the lability of methylated bases also leads to abasic sites. Furthermore, the modified bases are blocks to replicative polymerases and MNNG exposure leads to the formation of DSBs in DNA of cells exposed to it. Widtype and *recA* strains were exposed to MNNG in broth and glucose minimal medium and the results are shown in Fig. 5. The strains are sensitive in both media and MNNG appears more toxic in glucose minimal versus broth medium. It appears, therefore, that drug-resistance in minimal medium is associated only with BLM and is not a general phenomenon of DNA damaging agents.

### Detection of DNA breaks

**Figure 6:**
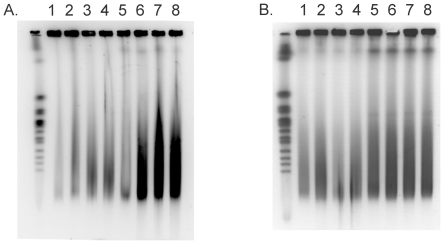
Pulse field gel electrophoresis of DNA from cells exposed, or not, to BLM. (A). Wildtype (lanes 1–4) or *recBCD* strains (lanes 5–8) growing in L broth were exposed to 0, 0.175, 0.35, and 0.70 µM BLM (lanes 1–4, 5–8) for 30 min before harvesting and processing. (B). The same as in A except that cells were cultivated in glycerol minimal medium.

We used pulsed field gel electrophoresis to detect DNA DSBs in cells after BLM exposure. The RecBCD exonuclease acts on DNA ends generated by DSBs [10] and so we used a *recBCD* strain to stabilize such ends. Wildtype or *recBCD* cells were grown in either L broth or glucose or minimal medium and exposed to various concentrations of BLM for 30 min, centrifuged and washed once to remove BLM, and then incubated for a further 30 and 60 min in respective growth medium. Samples of the washed and incubated cell population were removed, embedded in agarose and subjected to pulse field gel electrophoresis. The results are shown in Fig. 6. The wildtype strain, GM7330, growing in L broth, shows a dose-dependent increase in low molecular weight DNA after BLM exposure (Fig. 6A, lanes 1–4). Compared to the wildtype strain, the *recBCD* strain growing in L broth shows a dose-dependent increased accumulation of low molecular weight DNA after BLM exposure (Fig. 6A, lanes 5–8). In glucose minimal medium, however, there is no dose-dependent accumulation of low molecular weight DNA in either the wildtype or *recBCD* cells (Fig. 6B). These results indicate that, after BLM exposure, DSBs are detectable in cells growing in broth but not in cells growing in glucose minimal medium.

### Microarray analysis of treated cells

To gain insight on the mechanism of resistance of cells to BLM, total RNA was isolated from bacteria growing in either L broth or glucose minimal medium and challenged or not with 0.7 µM BLM. The microarray data from exponentially growing BLM treated cells was compared to that from untreated cells. The abbreviated results are shown in Table 1 and the complete data are in Tables S1 and S2.

**Table 1:** Increase in gene expression after challenge with BLM.

GM7330 (wildtype)				GM7661 (Δ (*recA-srl*)*306*::Tn*10*)			
L broth		MS		L broth		MS	
Gene	Fold increase	Gene	Fold increase	Gene	Fold increase	Gene	Fold increase
*recN*	15.0	*recN*	4.0	*nrd*	2.5	*rygA*	4.5
*umuD*	10.2	*sokC*	3.1	*yfaE*	2.4	*hycD*	3.1
*umuC*	6.2	*mltA*	3.0	b0423	2.1	*yjeT*	3.1
*sulA*	5.5	*dinD*	2.4	*nrdA*	2.1	*tnaL*	3.0
*oraA*	4.9	*sulA*	2.4	*dnaA*	2.1	*bglB*	2.9
*dinI*	4.1	*dinI*	2.4	*gltB*	2.1	b1011	2.5
*dinD*	4.0	*umuC*	2.2	*yigB*	2.0	*ygeW*	2.1
*dinB*	3.4	*napA*	2.1			b0941	2.1
*sbmC*	3.3	*proV*	2.0			b2209	2.1
*yebF*	3.3	*fdnH*	2.0			*ydfD*	2.1
*polB*	3.1	*tnaL*	2.0			*spoT*	2.0
*dinF*	3.0	*mutL*	2.0			*yibK*	2.0
*yebG*	2.8					*sgcC*	2.0
*uvrA*	2.7						
*ybiA*	2.7						
*recA*	2.6						
*yigN*	2.4						
*yjgL*	2.2						
*yafP*	2.1						
*ybiB*	2.0						
*uvrB*	2.0						
*yeeA*	2.0						
*ruvA*	2.0						
*lexA*	1.9						
*dinG*	1.7						

The *lexA* and *dinG* genes are included in this table because they are part of the SOS regulon and close to the cut-off value of 2.0.

Wildtype bacteria exposed to BLM in L broth show a robust SOS regulon induction with the *recN* gene showing the greatest increase (Table 1). As expected, there is no induction of SOS genes in the *recA* strain in either media. In the *recA* strain growing in L broth and exposed to BLM, there is increased induction of the initiator of chromosome replication protein, DnaA; the ribonucleotide reductase (*nrd*) genes, and its associated cofactor ferridoxin gene, *yfaE*. These results suggest that there is increased initiation at *oriC* and increased levels of deoxyribonucleoside triphosphates in BLM-stressed *recA* cells growing in L broth.

In glucose minimal medium, SOS induction occurs in wildtype cells exposed to BLM but the fold induction and the number of expressed SOS genes is less than in L broth (Table 1). The *sokC* gene encodes a small regulatory RNA that indirectly blocks translation of the HokC toxic membrane polypeptide. The *mltA* gene encodes a membrane-bound murein hydrolase and the *napA* gene encodes a nitrate reductase. The *proV* gene produces a high affinity glycine transporter and the *fdnH* gene encodes a subunit for formate hydrogenase N, an integral inner membrane protein. It is not clear how increased expression of any of these non-SOS genes can produce BLM resistance.

In the *recA* bacteria in glucose minimal medium and exposed to BLM, the gene with the largest fold increase is *rygA* (*omrA*), which encodes a small non-coding regulatory RNA (Table 1). Together with OmrB, another small RNA, OmrA may regulate outer membrane composition in response to environmental stress. The *hycD* gene product is part of the formate hydrogenlyase complex; the *tnaL* gene encodes tryptophanase; the cryptic *bglB* gene encodes phospho-beta-glucosidase; b1011 (*rutB*) is required for utilization of pyrimidines; b0941 encodes a fimbrin subunit; b2209 (*eco*) encodes a serine protease inhibitor; the *spoT* gene product is a ppGpp pyrophosphohydrolase; and *sgcC* encodes a predicted phosphotransferase . The functions of the *yjeT*, *ygeW*, *ydfD*, *yibK* genes are not known.

There was no overlap (other than tryptophanase) between wildtype and the *recA* strains in genes showing increased expression after BLM treatment in glucose minimal medium. Genes showing greater than a two-fold decrease in transcription were few and not informative. The microarray analysis did not, therefore, reveal a transcriptional mechanism responsible for BLM resistance. It should be noted that the array data represent only a single set of mRNA samples.

## Discussion

### DNA breaks in BLM-exposed cells growing in broth

For *E. coli* cells growing in broth, exposure to BLM results in the formation of DSBs. We base this conclusion on the detection of such breaks by pulse field gel electrophoresis; by the induction of the SOS regulon; and by the requirement for homologous recombination for cells to survive BLM challenge. The requirement for the RecBCD pathway of homologous recombination, and not the RecF pathway, is consistent with the preferential ability of this pathway to repair DSBs [10]. Single-strand breaks are also formed based on the sensitivity of DNA ligase mutant strains. These results for cells growing in broth were expected based on previous studies with other cytotoxic agents [13].

While this work was in progress, Nichols et al. [14] published the results of a high throughput method to determine *E. coli* phenotypes using the response of the Keio collection strains to various drugs including BLM. These studies were conducted in cells growing in rich (L) media. In general, the results of their study are in agreement with those reported here in terms of the requirement for recombination gene products (*recA*, *recC*, *recN*, *recG*) and DNA ligase. In addition, the Nichols et al. study identified *hfq*, *fis*, *rusA* and *sbcA* mutant strains as BLM sensitive as well as a large number of mutant bacteria affecting the structure/function of the cell envelope. The RusA and SbcA proteins are part of a prophage recombination system. The requirement for Hfq likely reflects a requirement for one or more small regulatory non-coding RNA molecules and their study identified *istR* and *gvcB* although additional RNA molecules are possible. The Fis protein is important to maintain nucleoid integrity. In sum, both studies indicate the importance of recombination in the resistance of *E. coli* cells growing in broth to BLM.

The results of the Nichols et al. study [14] also confirmed the data from two previous investigations. Girgis et al. [15] mutagenized *E. coli* with a transposon and selected for mutant strains with greater or less resistance to a panel of antibiotics including BLM. Becket et al. [16] screened the Keio collection [17] for sensitivity to various antibiotics. These determinations were carried out in L broth and, in general, the results from all three studies are in agreement.

The Nichols study did not identify a requirement for gene products involved in repair of oxidative damage to DNA. In this study, however, we showed that *xthA nfo* mutant bacteria were sensitive to BLM. This discrepancy is most probably because the single *xthA* and *nfo* mutant cells are not as sensitive as the double mutant.

### Sensitivity of *recG* and *recN* mutants strains to BLM

The sensitivity of *recG* and *recN* mutant strains growing in broth to BLM was confirmed. These mutant strains are not sensitive to other commonly used DNA damaging agents such as ultra-violet light or the methylating agents, ethyl- and methyl methane sulfonate [10]. BLM must cause a specific DNA lesion(s) that requires RecG and RecN and which is not produced by other commonly used DNA damaging agents. In the absence of RecBCD, *recN* mutant strains are sensitive to ionizing radiation, but not ultra-violet light, suggesting a role in repair of DSBs and/or oxidative damage through the RecF pathway [10], [18], [19]. Transcription of the *recN* gene is strongly stimulated during the SOS response [20], [21]. However, the function of the *recN* gene product remains unknown although it is a member of the SMC (structural maintenance of chromosomes) family of proteins.

RecG is a helicase that can translocate Holliday junctions but in the opposite direction to RuvAB [10], [22]. *recG* mutants are only slightly sensitive to ultra-violet light or X-rays but show increased sensitivity in a *ruvAB* mutant background [23]. Recently, Rudolph et al. [24] proposed that RecG prevents PriA mediated over-replication of UV-irradiated chromosomal DNA. In the absence of RecG, extensive replication occurs at chromosomal sites where replication forks have been inactivated. New forks are initiated through the action of PriA, which can load the DnaC protein, which in turn can load the replicative DnaB helicase. It is possible that such a “pathological cascade” may also occur in *recG* cells exposed to BLM. Alternatively, RecG might unwind persistent recombination intermediates that would otherwise compromise chromosome replication.

### DNA breaks in BLM-exposed cells growing in glucose minimal medium

In contrast to bacteria growing in broth, cells growing in glucose minimal medium were resistant to the cytotoxic effects of BLM. This result was not expected as other DNA-damaging agents are effective in both types of media. Single-strand breaks were formed in *E. coli* cells growing in minimal medium based on the increased sensitivity to BLM of ligase-deficient strains (Fig. 4). Alternatively, the requirement for DNA ligase in both broth and minimal medium might reflect the increased need for this enzyme as a result of base excision repair of lesions. There is no requirement for homologous recombination functions and only a weak SOS response in wildtype cells exposed to BLM. The sensitivity of the ligase-deficient strains would minimize, but not exclude, the possibility of reduced transport of BLM into the cell as an explanation for resistance.

The lack of BLM sensitivity for cells growing in minimal medium could be due to the reduced number of replication forks in such cells as compared to those grown in broth. Fewer forks would reduce encounters with lesions to form DSBs. This explanation would account for the observation of reduced number of DSBs, and reduced SOS induction in minimal medium growing cells. However, when the BLM dose was increased to cause substantial cytotoxicity in the wildtype (Fig. 3), there was no requirement for homologous recombination which is not consistent with replication fork breakdown and restart. Furthermore, cells growing in minimal medium were killed by MNNG, a chemical known to produce replication-blocking lesions, indicating that sufficient forks are present in bacteria growing in minimal medium.

BLM can produce DNA strand breaks in the test tube when oxygen and ferrous ion are supplied. It may not be necessary, therefore, for cells to metabolize BLM to an active form. Drug resistance through failure to activate BLM seems unlikely especially given the susceptibility of the DNA ligase-deficient mutants to BLM toxicity. Alternatively, cells can become resistant to BLM either by protein sequestration [25] or by having a cysteine peptidase to cleave BLM to an inactive form [2]. Either of these possibilities could explain BLM resistance of *E. coli* cells growing in glucose minimal medium but such proteins have not yet been identified. Alternatively, ferrous ions may be more available in fast growing cells than cells growing slowly; that is, there may be greater exchange of metal from stores into a cellular pool for use by active enzymes or BLM.

The control samples for the microarray analysis allowed us to compare the transcriptional profiles of cells growing in broth and glucose minimal medium. Transcription profiles of cells in the logarithmic phase of growth were compared to stationary phase cells. For cells growing in broth, 46 of the 100 most highly transcribed genes were those encoding flagella biosynthesis and chemotaxis. Others in the top 100 were tryptophanase, succinate dehydrogenase, and D-ribose binding and transport proteins. For cells growing in glucose minimal medium, the transcription profile was very different. There were only 19 genes encoding flagella biosynthesis and chemotaxis in the top 100. The most highly expressed genes (6 in the top 12) were for maltose binding and transport. There were 21 genes in the top 100 associated with Fe binding and transport. A putative zinc protease gene, *pqqL*, was the 93rd most highly transcribed gene. However, the *pqqL* mutant strain in the Keio collection [17] behaved like the GM7330 wildtype strain in its survival after BLM challenge (data not shown). Unfortunately, the array transcriptional data do not allow identification of the mechanism of BLM resistance.

Previous studies investigating the action of BLM on *E. coli* in L broth identified a putative protease that when inactivated increased toxicity [14]–[16]. We confirmed that the mutant strain (*yfgC*) encoding this protein was more sensitive than wildtype when cultivated in L broth but was as resistant as wildtype when tested in glucose minimal medium (data not shown).

Further studies could include a screen of the Keio collection for cells sensitive to BLM on glucoase minimal medium. Alternatively, or in addition, mutagenized cultures could be screened for this property.

## Materials and Methods

### Bacterial strains

The strains used are derived from GM7330 (except for the *lig-7* (Ts) mutant) and the most important are described in Table 2. The full genotypes of other GM7330 mutant strains have been described elsewhere [12], [26].

**Table 2:** Some of the *E. coli* K-12 strains used in this study.

Strain	Description	Source of mutation/strain
GM7330	F^−^ Δ(*lacY-lacZ)286* (ϕ80*d*IIΔ *lacZ9*) *ara thi* (?)	KS418
GM7332	GM7330 Δ*recG263*::Kan Met^−^	R.G. Lloyd
GM7338	GM7330 *ruvC53 eda51*::Tn*10* Δ*recG263*::Kan Met^−^	GM7332
GM7346	GM7330 Δ*recBCD*::Kan	K.C. Murphy
GM7390	GM7330 Δ*ruvABC*::Cam	R.G. Lloyd
GM7661	GM7330 Δ (*recA-srl*)*306*::Tn*10*	A.J. Clarke
GM7663	GM7330 Δ*recN*::Tet	A. Poteete
GM8492	GM7330 Δ*xth*A::Kan Δ*nfo*::Cam	R. Cunningham
KS418	F^−^ Δ(*lacY-lacZ)286* (ϕ80*d*IIΔ *lacZ9*) *ara thi* (?) Met^−^	B. Konrad
N2668	*lig-7* (Ts) *rpsL*	M. Gottesman

Doubling times for the wildtype strain was 36 min in L broth, 54 min in glucose minimal salts, and 84 min in glycerol minimal salts.

### Media

The rich medium used was Luria (L) broth which consists of 20 g tryptone, 10 g yeast extract, 0.5 g NaCl and 4 ml 1 M NaOH per l, and solidified when required with 16 g agar (Difco). Minimal medium contained minimal salts as described by Davis and Mingioli [27] supplemented with 0.4% glucose or glycerol and 0.2 µg thiamine per ml. Ampicillin and rifampicin were used at a concentration of 100 µg/ml, kanamycin at 20 µg/ml, tetracycline and chloramphenicol at 10 µg/ml and carbenicillin at 50 µg/ml.

### BLM toxicity assay

Cells in L broth or minimal medium with glucose or glycerol as carbon source were grown to an OD_600_ = 0.2–0.3 and exposed to BLM (Bleomycin sulfate, Sigma-Aldrich) for 30 min after which they were diluted in minimal salts and plated on L media and incubated overnight at 37°C. There was no difference in viable counts from the cells exposed to BLM in minimal medium when plated on rich medium or minimal medium plates. For the temperature-sensitive (Ts) *lig-7* cells, cultures were grown at 30°C and then shifted to 42°C for 30 min before adding BLM. In the absence of BLM, there was no decrease in survival after a 60 min incubation at 42°C.

### Preparation of plugs and Pulse field gel electrophoresis (PFGE) migration

We have used the procedure described by Seigneur et al. [28] with modifications [29]. Cells were cultivated in L broth or minimal medium with glycerol or glucose as carbon source and grown at 37°C to an OD_600_ = 0.2–0.3 and exposed to BLM for 30 min after which the cells were harvested by centrifugation, washed once with minimal salts, twice in SE buffer (75 mM NaCl, 25 mM EDTA, pH 7.4) and resuspended in 160 µl of distilled water. The cells were mixed with an equal volume of 2% low melt agarose (in 0.5×TBE buffer), distributed in 60 µl portions in molds and left for a few minutes at 4°C until set. The plugs were incubated overnight at 56°C in 1 ml LE buffer (1% N-laurylsarcosine, 0.5 M EDTA, pH 9.6) with proteinase K (0.5%) [30]. The plugs were then washed three times with TE buffer and stored in TE buffer at 4°C. Portions of each agarose plug were loaded into the wells of 1% agarose gels (Seakem Gold Agarose) and sealed with 1.0% low melt agarose and were subjected to electrophoresis at 14°C for 24 h at 6 V/cm in 0.5× TBE, with an initial switching time 60 s and final switching time of 120 s in a BioRad CHEF-DR II apparatus. Plugs containing the chromosomes of the yeast *Saccharomyces cerevisiae* were routinely used as molecular weight standards (New England Biolabs). For each of the PFGE figures, the experiment was done at least three times and a representative figure is shown.

### Gene Chip arrays

The procedure was that recommended for the Affymetrix Gene Chip *E. coli* Genome 2.0 Array. Cells were cultivated and exposed or not to 0.7 µM BLM as described above and total RNA was isolated using the MasterPure RNA Purification kit (Epicentre Technologies). Further steps in the procedure were carried out at the UMass Medical School Genomics Core. Random primers (Life Technologies) were annealed to 10 µg of the RNA and SuperScript II Reverse Transcriptase used for cDNA synthesis. Residual RNA was removed by alkaline hydrolysis and the cDNA was purified using a Qiagen MinElute PCR Purification Kit. The cDNA was fragmented with DNAse I (Amersham Biosciences) in One-Phor-All buffer (Amersham Biosciences) and end-labeled using Terminal Deoxynucleotidyl Transferase (Promega) and GeneChip DNA Labeling Reagent (Affymetrix). The end-labeled RNA was hybridized to an Affymetrix GeneChip *E. coli* Genome 2.0 Array in a GeneChip Hybridization Oven 320 at 45°C for 16 hours, then washed in the Affymetrix Fluidics Station 450 and scanned in the Affymetrix GeneChip Scanner 3000. Full details of this procedure are available at the Affymetrix website (www.affymetrix.com). The results from the hybridization were exported as Microsoft Pivot files and imported into Microsoft Excel for sorting. The arrays were done only once as the results were not informative as to the mechanism of BLM resistance (see Results).

## Supporting Information

Table S1 Gene Chip 2.0 values from GM7330 cells exposed or not to BLM in L broth and minimal medium.
(XLSX)

Table S2 Gene Chip 2.0 values from GM7661 cells exposed or not to BLM in L broth and minimal medium.
(XLSX)
